# Parliament and Publications

**Published:** 1911-06-03

**Authors:** 


### Parliament and Publications.

### ST. ANDREW'S AMBULANCE ASSOCIATION.

A Bill has been introduced in the House of Commons to confirm a provisional order to authorise the St. Andrew's Ambulance Association to transfer a portion of the Red Cross Fund, held by that association, to the Scottish Branch of the British Red Cross Society.

### GUN DEAFNESS.

Replying to Mr. Fitzroy, Mr. McKenna stated that there were many cases of officers and men in the Royal Navy whose hearing had been impaired through gun deafness. Tests had been carried out upon various preventatives, but opinions differed as to their relative efficiency. An ear paste is already prepared and issued where demanded.

### MR. BALFOUR ON SANATORIA.

In the course of the debate on the financial resolutions, Mr. Balfour made some observations on that part of the Chancellor's scheme which furnishes large capital sums for the building of sanatoria throughout the country. He expressed the opinion that perhaps there had been in the public mind an undue or slightly exaggerated enthusiasm with regard to the sanatorium method of dealing with tuberculosis. He was not quite sure that the most recent investigations into the results of treatment in sanatoria bore out all the hopes that they were at one time prepared to entertain in regard to it. He certainly took a sanguine view as to the treatment of tuberculosis, but when they came to such large capital sums as those dealt with by the Chancellor of the Exchequer, he hoped the Chancellor would bear in mind that while it is possible to waste money upon vast, permanent, and expensive structures, it is not really possible to waste money if it was devoted judiciously to scientific medical investigations into the causes and cure of disease. Mr. Lloyd George, who followed, said he did not think sanatoria by any means the last word, but they were a stage on the road. He did not think even that if sanatoria turned out to be a crude idea, the money would be wasted, because what would happen would be that in the case of a great many patients—twenty thousand or thirty thousand it might be—they would have doctors who would be specialists in this particular kind of disease. They would be watching it day by day in its development, and they were bound to get to know far more about it than at the present time. In the end, especially if there was a fund of this kind set up, they might be able to find something far more perfect in the way of treatment than is known up to the present time.

### ROYAL ARMY MEDICAL CORPS.

Replying to Mr. Sandys, who asked whether the strength of the Royal Army Medical Corps had been reduced since 1906 by 488, Mr. Ackland replied that the establishment of the Royal Army Medical Corps, warrant officers, non-commissioned officers and men, in 1906 was 4,189, and the present establishment is 3,947, showing a decrease of 242. Of these 190 were men specially enlisted for one year in 1906 to augment the reserve and thirty-eight were reduced from South Africa in 1907, leaving a balance of fourteen for reduction in various stations since 1906.

